# Pedagogical health – a holistic perspective on promoting children’s and students’ well-being in school

**DOI:** 10.1080/17482631.2026.2740237

**Published:** 2026-09-29

**Authors:** Ann-Louise Ljungblad, Lisa Hellström

**Affiliations:** a Department of School Development and Leadership, Malmö University, Malmö, Sweden

**Keywords:** Mental health, physical health, social health, existential health, pedagogical health, school health promotion, interprofessional collaboration, qualitative research

## Abstract

**Purpose:**

Gloabally, young people are experiencing increasing ill health. Since school health promotion often lacks an interprofessional language, this study strives to open up a global conversation by introducing *Pedagogical Health in School (PHIS).* This serves as a novel pedagogical lens to bridge individual-level well-being with school-level structures, providing a shared conceptual base for interprofessional collaboration.

**Method:**

Drawing on qualitative data from preschool through upper secondary school, the study explores the umbrella concept of *pedagogical health,* encompassing four health foundations: mental, physical, social, and existential health, and offering *a holistic perspective* on children’s and students’ well-being in school settings.

**Results:**

The *primary contribution* is the identification of *52 candidate indicators* manifesting across the four foundations in practice. These candidate indicators translate broad health concepts into observable phenomena, offering school staff a concrete, shared basis for interprofessional dialogue and action.

**Conclusions:**

The article proposes pedagogically grounded definitions for each foundation to support coordinated health-promoting work. PHIS represents a significant advancement in the field of school health promotion by providing *a holistic, pedagogically grounded perspective* that aligns practice with Sustainable Development Goals (SDG 3 and 4). By integrating well-being into pedagogical settings, PHIS strengthens schools’ capacity for proactive and sustainable health promotion.

## 1. Introduction

Since the turn of the millennium, there has been growing recognition that young people are experiencing increasing levels of ill health across many regions, with rising mental, physical, social, and existential challenges (United Nations Department of Economic & Social Affairs, 2025). The World Health Organisation (World Health Organisation, 2025a) indicates that around one in seven young people aged 10 to 19 experience mental health challenges. Cross-national studies suggest that approximately one-third of adolescents report psychological and somatic symptoms (Schrijvers et al., 2024). Reports from various countries indicate that contemporary youth face more challenges to their well-being than previous generations (Cosma et al., 2023). These developments are linked to societal transformations and increasing demands on young people, which may amplify stress and feelings of inadequacy (Bor et al., 2014; Esposito & Perez, 2014). This trend highlights the urgent need for research examining inequalities in young people’s health (Beckwith et al., 2024) and the child’s right to health (United Nations General Assembley, 1989).

Health is defined by WHO as “a state of complete physical, mental, and social well-being and not merely the absence of disease or infirmity”, reflecting a holistic view of health. Building on this perspective, existential health has also been emphasised as an important foundation. Education plays a pivotal role in shaping children’s health and well-being (United Nations Department of Economic & Social Affairs, 2025). Initiatives such as the European Network of Health Promoting Schools (ENHPS) aim to create healthy school environments and support students’ holistic development (Barnekow Rasmussen & Rivett, 2000). To achieve these goals, structured frameworks and resources for both educators and school staff are essential. The current study presented in this article contributes to this field of school-based health promotion.

WHO and United Nations Educational, Scientific and Cultural Organisation (WHO & UNESCO, 2021) propose global standards for health-promoting schools, including indicators to support data-informed decision-making across system levels. However, a knowledge gap remains: there is a need for indicators that connect the individual and school levels and can be used by all school staff in interprofessional collaboration. Moreover, research shows that health professionals and educational staff often lack a shared language for understanding health, which hampers coordinated and sustainable practices (Beckman et al., 2023; Simovska & McNamara, 2015). Without such a common language, it becomes difficult to coordinate efforts, evaluate interventions, and integrate health promotion into everyday school practices.000

This article contributes a pedagogically grounded perspective—*pedagogical health* (Hellström et al., 2024)—which helps address the identified knowledge gap by connecting individual well-being with school level structures. Based on empirical research, the aim is to explore pedagogical health across four foundations: mental, physical, social, and existential health. The research is guided by two questions: (RQ1) What candidate indicators within each health foundation signal a child’s or a student’s health or ill health? (RQ2) In what ways can these candidate indicators on an individual level inform pedagogical definitions within each health foundation to support interprofessional collaboration at a school level?

Given the increasing levels of ill health among the younger generation, this article adopts a proactive, sustainable, health-promoting perspective, embracing a holistic approach to understanding and enhancing children’s and students’ well-being in school contexts. This approach aligns with the United Nations Sustainable Development Goals—particularly *SDG 3 (Good Health and Well-being)* and *SDG 4 (Quality Education)*.

## 2. State of the art—children’s well-being and ill health

Children’s health requires a holistic approach, as it encompasses interconnected mental, physical, social, and existential foundations. According to World Health Organisation (2025b), *mental health* includes both mental well-being and mental ill health, such as psychological difficulties and psychiatric conditions. Mental health is a state of mental well-being that enables people to cope with the stresses of life, realise their abilities, learn and work well, and contribute to their community. It has intrinsic and instrumental value and is a basic human right.

Globally, research shows that mental health problems affect 10-20% of children and adolescents, and the prevalence in low- and middle-income countries is comparable to that in high-income countries (Kieling et al., 2011). Studies further underscore the importance of actively supporting youth mental health, as most mental disorders begin during adolescence and are associated with challenges such as lower educational attainment, substance use, and violence, as well as elevated rates of self-harm and suicide (Patel et al., 2007). Some argue that the increasing levels of mental health concerns among adolescents can be explained by a growing number of stressors in the school environment (Högberg et al., 2020), and enhancing student wellbeing is becoming a key focus worldwide (Schmitz, 2024). Risk factors include social exclusion, test anxiety, and academic pressure such as grades (Fassl et al., 2025). In contrast, students’ well-being is positively associated with factors such as feeling accepted by teachers, a supportive classroom climate, a positive ability self-concept, and an overall positive school mood (Eckert et al., 2025). Adolescents typically describe, make sense of, and communicate their mental health difficulties using everyday language—such as sadness, loneliness, shame, boredom, anger, and a sense of defeat (UNICEF, 2022). A systematic review of school-based mental health interventions (Spencer et al., 2025) provides evidence that selective and indicated interventions can improve students’ mental health and well-being, particularly by reducing psychological distress, anxiety, and related symptoms. The findings highlight the importance of early-stage interventions, which may strengthen self-esteem and coping strategies that foster resilience. However, intervention effects vary depending on intensity and duration, and further research is needed to identify which characteristics support long-term sustainability (Spencer et al., 2025). At the same time, a lack of shared terminology in mental health research complicates coordinated efforts (Choi, 2024). In summary, while the literature highlights a clear link between school-related stressors and declining mental well-being, a significant challenge remains in the *lack of shared terminology* and the fragmented nature of interventions. This underscores the need for a *pedagogical lens* that can translate medical mental health concepts into practice-recognisable expressions within the classroom to support early-stage, sustainable interventions.

*Physical health* is closely linked to physical activity, which supports not only physical but also mental and social health (Poitras et al., 2016; Robinson et al., 2018). However, as lifestyle-related risk factors emerge early in life and patterns of physical activity tend to persist into adulthood (Jones et al., 2013), childhood represents a critical window for establishing health-promoting behaviours with long-term implications that may enhance future health (Fernandez-Jimenez et al., 2018). Globally, 80% of young people are insufficiently active, and many adolescents engage in two or more hours of daily recreational screen time (van Sluijs et al., 2021). Sedentary patterns, particularly screen use and TV viewing, are linked to adverse health outcomes, partly due to associated unhealthy dietary habits (Carson et al., 2016; Hobbs et al., 2015). Sedentary lifestyles negatively affect physical health, particularly sleep quality, while many children and adolescents do not achieve sufficient sleep (Chaput et al., 2016; Hartstein et al., 2024). These patterns may have long-term consequences for well-being, learning, and academic achievement (Norell-Clarke & Hagquist, 2017). Synthesising these findings, it is evident that the sedentary trend among youth necessitates a shift from viewing physical health as an individual responsibility to a *collective pedagogical commitment*. By integrating physical activity into the school’s daily routines and learning environments, schools can address the critical window for establishing long-term health-promoting behaviours that are essential for both well-being and academic achievement.

When it comes to children’s and students’ *social health* in educational settings, international relational research (Aldridge & McChesney, 2018; Krane et al., 2016; Martin & Dowson, 2009; Roorda et al., 2011) consistently demonstrates that secure and positive teacher–student relationships (1) create safety, well-being, and health for students, (2) strengthen students’ self-confidence and their belief in their ability to learn, (3) promote students’ learning and school success, and more specifically, (4) support their social and emotional development and academic performance. Such research, which places relationships at the core of education, shows that when relationships and interactions are intentionally prioritised and strengthened across the school years, students’ learning, emotional, and social outcomes improve (Pianta, 2026). Empirical research exploring what happens in-between human beings provides an important foundation for well-being and is highly relevant for educational practice, where trustful relationships between teachers and students, as well as between school leaders and staff, form the basis for sustainable relationships in education (Ljungblad & Sidorkin, 2026). Taken together, the research demonstrates that social health is the very *foundation of the educational encounter,* rather than merely a school attendance byproduct. A synthesis of these relational perspectives suggests that sustainable health promotion requires a systematic approach to mapping and fostering secure attachments, ensuring that every student is seen and part of a meaningful social context throughout their entire health pathway.

Social health and well-being are closely linked to *existential health*, which relates to experiencing meaning in life; being in relation to oneself, others, and nature; and feeling part of something greater. Supporting well-being in the face of existential concerns means finding a place to rest—a balance between movement and stillness that allows reflection on one’s life story to enhance self-understanding (Lundvall et al., 2022). When such concerns become overwhelming, support from healthcare professionals is needed and should be open and centred on the young person’s life story. One visible manifestation of these challenges is rising school absenteeism, where increasing absence may signal underlying struggles with well-being and belonging (Kearney et al., 2023). More broadly, research shows a strong connection between health and education (Ventriglio & Bhugra, 2023). To conclude, the literature suggests that existential health serves as the ‘resting place’ for a student’s identity amidst modern pressures of performance and judgement. The synthesis of these existential concerns points toward a critical need for *safe pedagogical forums* where young people can process life’s fluctuations, thereby moving from a state of mere performance to a state of meaningful belonging and resilience.

Over recent decades, health promotion research has shifted from pathology-oriented prevention models to holistic approaches that strengthen well-being and life skills (Terjesen et al., 2004). For example, universal school-based resilience programs draw on decades of research, emphasising the interplay among psychological, familial, social, and institutional dimensions. These interventions aim to enhance protective factors that buffer stress and promotive factors that support positive development (Tasijawa & Siagian, 2022). Consequently, schools have a unique potential to promote health and well-being, as they reach nearly all children and adolescents (Seligman et al., 2009).

## 3. School health promotion frameworks

Health and health habits are established early in life (Marmot and Bell, 2012), making early interventions across the home, preschools, and schools essential (WHO & UNESCO, 2021). As institutions reaching all children, schools hold a unique position in addressing health challenges, underscoring the need for sustainable school improvement approaches (Kearney & Graczyk, 2020). However, evidence suggests that many schools remain uncertain about how to respond to the complex and evolving needs of today’s youth (Ghose et al., 2024; Miller & Thabrew, 2024). Contemporary school-based health promotion is understood as a holistic, multidimensional process aligned with World Health Organisation (1986) definition of health as encompassing physical, mental, and social well-being, with additional emphasis on existential foundations such as meaning and identity. Schools thus function not only as academic institutions but also as environments shaping students’ overall well-being through daily interactions, pedagogical practices, and organisational structures.

Several models guide school health promotion. Bronfenbrenner’s ecological theory (Bronfenbrenner, 1979) highlights how health is shaped through interactions across environmental layers, indicating that health promotion must address social and structural conditions. Seligman’s PERMA model adds a complementary perspective, conceptualising flourishing through Positive Emotion, Engagement, Relationships, Meaning, and Accomplishment, aligning with multidimensional understandings of health. Research shows that schools can foster these elements through intentional pedagogy, supportive relationships, and well-designed learning environments (Seligman, 2011). The WHO and UNESCO Health Promoting School (HPS) framework (WHO & UNESCO, 2021) provides a system-level approach, aligning with ecological and positive psychology perspectives by emphasising supportive relationships, engagement, and belonging. Evidence shows that HPS interventions can improve both health and educational outcomes (Langford et al., 2015). Similarly, whole-school approaches stress that health promotion is most effective when integrated across curriculum, culture, leadership, and community partnerships, contributing to sustainable and equitable health outcomes (Edwards et al., 2024).

### 3.1. Conceptual positioning: pedagogical health as an analytical lens

Established frameworks within health-promoting schools (WHO & UNESCO, 2021), whole-school approaches to well-being, and school climate research provide an important system-wide orientation. However, they often lack analytically grounded tools to capture how children’s and students’ health and ill-health become visible in everyday pedagogical practice. As these frameworks primarily emphasise organisational structures, programmes, or general environmental conditions, they offer limited analytical precision at the individual level of children’s and students’ lived health experiences as encountered by school staff in daily work. Despite the strong theoretical foundations of existing frameworks, a key challenge remains: the absence of a shared professional language among school staff. Teachers, student health teams, administrators, and support staff often operate with different conceptual silos, leading to fragmented practices (Beckman et al., 2023; Simovska & McNamara, 2015). This study aims to bridge this gap.

In this article, *pedagogical health* is introduced not as a new intervention model or framework but as a pedagogical lens that connects holistic health perspectives with observable pedagogically phenomena. The concept of pedagogical health functions as a second-order construct, translating broad health concepts into professionally recognisable units that school staff can identify and discuss in their daily work. By lowering the level of abstraction, the PHIS perspective makes holistic well-being analytically accessible within the pedagogical life of schools, supporting coordinated considerations across pedagogical, social, and medical domains while remaining sensitive to each child’s unique health profile.

In line with WHO and UNESCO frameworks for health-promoting schools, this Swedish study strives to open up a global conversation by adopting the *novel perspective—Pedagogical Health in School (PHIS)*. This serves as *a pedagogical lens* to bridge the individual level of children’s and students’ health and well-being with school-level structures, providing a shared conceptual base for interprofessional collaboration to support children’s well-being. Accordingly, this article introduces the concept of PHIS as a holistic, pedagogically grounded perspective designed to strengthen schools’ capacity for interprofessional, proactive, and sustainable health-promoting and preventive work.

## 4. Method

### 4.1. Study design and context

This article is based on a secondary, in-depth qualitative analysis of previously collected focus group data from a large municipal research collaboration in Sweden (Hellström et al., 2024). The study was conducted in the City of Malmö, a highly diverse and multicultural urban environment characterised by complex socio-economic conditions, migration flows, and substantial variation in educational needs across schools. The city is therefore an important and illustrative example when exploring the complex conditions surrounding children’s well-being and ill health. The study was carried out as commissioned research on behalf of the City of Malmö.

The original project explored how health-promoting work was organised and experienced across educational levels—from preschool to upper-secondary school—within the city’s three school administrations. The aim was to investigate schools’ initiatives and methods related to children’s health and to examine how school staff perceive and experience health-promoting practices. Hence, from a pedagogical perspective, the study explored professional experiences and perspectives related to children’s and students’ health in everyday school practice, following the child’s health path across the city’s educational system.

The dataset consists of 17 focus group discussions with 66 participants, addressing issues related to children’s and students’ health and well-being in the school context. That the available financial resources only permitted the study to capture adults’ experiences—and not students’ voices—constitutes a limitation in fully understanding the phenomenon studied. There was, nonetheless, a clear awareness within the project that such a follow-up study would be desirable. Due to time constraints, leisure centres, Special Needs Schools, and students were not interviewed. A reference group consisting of professionals with diverse competencies from the city assisted the researchers in recruiting participants. The participants represented a wide range of professional roles, including preschool teachers, primary and secondary school teachers, Special Educational Needs (SEN) teachers, student health team members, educational developers, principals, and municipal specialists. All professional groups involved in the school’s health-promoting work were represented in the study, ensuring broad cross-professional participation.

Audio-recorded interviews were conducted at different schools, each time in a designated, uninterrupted room selected to ensure a confidential interview environment. Two of the researchers conducted each interview. Each interview lasted approximately 50–60 minutes, resulting in a total of 851 minutes of audio-recorded data. All interviews were fully transcribed. In the analysis of the original study, a thematic analysis (Braun & Clarke, 2006) was conducted. The three researchers brought complementary expertise in health promotion, special needs education, and pedagogical school research, providing a broad and multifaceted analytical perspective. The results revealed a consistent pattern: professionals across different roles reported similar experiences of increasing ill health among children and young people. This concordance extended not only within and between the various professional groups, but also across educational levels—from preschool through compulsory school to upper-secondary education. Notably, participants described a largely shared understanding of how children’s ill health manifests within a pedagogical context. The results of the original study revealed four overarching themes: (1) the need for an interprofessional language for health-promoting work, (2) gaps in collaboration between organisations and professions, (3) the importance of interpersonal relationships, and (4) the working environment of children and students. An in-depth critical analysis further revealed that the support available in schools does not lead to equitable outcomes for individual students, and that the organisational structures governing student health work are not sustainably established. The original study concludes that the schools’ health-promoting work spans four foundations of children’s and students’ health—mental, physical, social, and existential—which are described under the umbrella concept of *pedagogical health.*

#### 4.1.1. Human ethics, informed consent and consent to participate

According to institutional and national guidelines in Sweden, formal ethical approval was not required for this study as it did not involve the collection of sensitive personal data or physical interventions. The research adheres to the national regulations stipulated in the Swedish Ethical Review Act (SFS, 2003:460), available via the Swedish Ethical Review Authority, and complies with the ethical guidelines outlined by the Swedish Research Council. Prior to participation, all participants received written and verbal information about the study, including its purpose, procedures, confidentiality measures, and their right to withdraw at any time without penalty. Written informed consent was obtained from all participants before participation in the study. While the interviews touched upon broad health themes, participants were explicitly informed that the study did not seek to collect any individual sensitive data. The analysis focused strictly on the participants’ professional observations and shared pedagogical experiences. Consequently, formal ethical approval was not required under the Swedish Ethical Review Act (SFS, 2003:460).

### 4.2. Analysis

The decision to conduct a secondary analysis was based on the untapped depth of the original dataset, which contained rich, cross-professional descriptions of children’s daily health manifestations that exceeded the scope of the primary study. This existing material provided an ideal foundation for RQ1 and RQ2, as it captured spontaneous professional observations of health indicators before they were theoretically categorised through the PHIS lens. In the second study, presented in this article, a renewed and in-depth deductive qualitative content analysis (Elo & Kyngäs, 2008) was conducted by the two authors of the article. The aim was to explore *pedagogical health.* This concept encompasses four health foundations—mental, physical, social, and existential health—and offers a holistic perspective on promoting and understanding children’s and students’ well-being in school. These foundations were also examined in relation to how all school staff can work with them in an interprofessional manner. The present study applies a pedagogical perspective to children’s health. Here, pedagogy refers to how schools create opportunities for unique children to emerge, express their own voices, and flourish (Ljungblad, 2021). Additionally, the analysis is guided by the principles of the child’s best interests (United Nations General Assembley, 1989).

In the preparation phase of the deductive analysis, which is often used when researchers wish to re-examine existing data **(**Elo & Kyngäs, 2008), the two researchers selected the following theoretical unit of analysis: four theoretical descriptions of health, each representing a distinct foundation of health:

(1) *Mental health* is defined as a state of well-being in which the individual realises their abilities, copes with the normal stresses of life, can work productively and fruitfully, and contributes to their community (World Health Organisation, 2005).
(2) *Physical health* is expressed in terms of overweight and obesity, sexual health, lifestyle habits such as physical activity, sedentary behaviour, sleep habits, use of alcohol, drugs, and tobacco (The Public Health Agency of Sweden, 2024).
(3) *Social health* refers to the quality and depth of one’s relationships. It extends beyond small talk or social media; social health is about forming genuine, meaningful connections. These relationships offer emotional support and provide a sense of belonging (Holt-Lunstad, 2024).
(4) *Existential health* relates to experiencing meaning in life, being in relation to self, others, and nature, as well as feelings of coherence, security, and being part of something greater (The Public Health Agency of Sweden, 2025).

Based on the analysis of the original study, both researchers possessed a deep understanding of the empirical material. In the second study, a deductive analysis made it possible to distil words and expressions into categories within each health foundation that share a common meaning. The initial phase of the in-depth analysis involved a systematic colour-coded categorisation system, where each of the four health foundations was assigned a specific colour to ensure analytical consistency across the 17 focus group transcripts. This internal documentation served as an audit trail, enabling the researchers to verify the progression from meaning units to candidate indicators and ensuring that each unit remained grounded in its respective theoretical foundation. All analytical decisions were cross-verified through continuous dialogue, establishing a robust consensus-based structure for the findings. The analysis followed a structured, multi-step procedure:

(1) It began with a close reading of the transcripts to identify *meaning units,* using a distinct colour for each of the four health foundations to ensure analytical consistency. These meaning units consisted of words, sentences, or longer excerpts that directly related to the four health foundations and the study’s analytical aim.
(2) Once identified, the frequently occurring meaning units were condensed to preserve their core content while reducing the volume of text.
(3) The condensed meaning units were then coded as descriptive labels**—**candidate indicators**—**that captured their manifest meaning. These candidate indicators illustrate concrete aspects of children’s health as described by school professionals.

One researcher assumed primary responsibility for the initial step (1) of the analysis—*identifying meaning units* using one colour for each health foundation. These meaning units were then verified by the second researcher, with no disagreements occurring. Steps (2) and (3) were conducted jointly, during which the analytical decisions within each health foundation were documented. Within each of the four foundations, the candidate indicators were examined to identify recurring content and commonalities. These health foundations served as the analytical framework guiding the organisation of the material. The categorisation was therefore theory-driven but grounded in the explicit expressions of the participants. The analysis deliberately remained at a manifest level of interpretation, focusing on what was explicitly stated rather than developing higher-order themes or latent meanings. This ensured methodological coherence with the study’s aim of identifying candidate indicators (RQ1). The deductive analysis was bounded at this level in the exploration of the theoretical concept *pedagogical health*, highlighting the need for future studies that draw on students’ experiential perspectives to enrich and extend the theoretical conceptualisation. To conclude, this structured procedure enabled an *analytical progression* from empirical findings to conceptual propositions. The identified candidate indicators function as second-order constructs that bridge individual-level health expressions with school-level organisational structures, effectively lowering the level of abstraction in global frameworks. This process culminated in a conceptual synthesis, viewed through a pedagogical lens, to derive the new proposed pedagogical definitions (RQ2).

#### 4.2.1. Analytical status of candidate indicators

The analytical aim of the study was not to generate an abstract theoretical model. Instead, based on school staff’s experiences the analysis focused on identifying frequently recurring meaning units that operate as pedagogically observable candidate indicators of children’s and students’ health and ill health. Grounded in manifest expressions articulated by participants, these candidate indicators represent second-order analytical constructs. They are analytically refined through their recurrence, cross-professional recognisability, and relevance across educational levels. Hence, the candidate indicators signal conditions and processes that support or undermine pedagogical health within everyday school contexts. The analysis deliberately remained at a manifest level of interpretation to preserve the immediacy and professional validity of the indicators. Rather than constituting a limitation, this analytical choice reflects the study’s purpose to develop candidate indicators that can function as shared reference points in interprofessional collaboration. These candidate indicators translate broad health concepts into analytically coherent and professionally recognisable units that school staff can identify, discuss and act upon in practice. Furthermore, the candidate indicators can support early identification of health-related vulnerabilities and resources in children’s and students’ unique health profiles. Below, an analytical example is presented to illustrate the progression from meaning unit to candidate indicator (Table I).

**Table I.** The table illustrates the stepwise progression of the deductive qualitative content analysis. (1) *Identification of meaning units:* Extracting relevant expressions from the interview transcripts. (2) *Condensation into candidate indicators:* The process where meaning units (e.g., ‘Students are being judged frequently’) are condensed and refined into a manifest *candidate indicator* (‘Daring to speak with your unique voice’). The *Analytical function* then demonstrates how this candidate indicator serves as a shared reference for identifying vulnerabilities in students’ existential health within a pedagogical environment.

*Interview excerpt*	“The feeling of being judged in many different contexts, at school and during their free time… It’s something you can’t really get away from nowadays… The matter of daring to speak is closely linked to the fear of failing. I think it’s important to get the chance to practice and maybe feel that it’s OK to fail.” (Teacher compulsory school)
*Meaning unit*	Students are being judged frequently in school. High demands on perfectionism. They do not dare to make mistakes.
*Candidate indicator*	Daring to speak with your unique voice.
*Analytical function*	This candidate indicator captures a core of existential health by linking students’ emotional experiences of being constantly judged to pedagogical learning conditions. Across the interviews, similar expressions recur among different professionals’ groups and educational levels, enable the candidate indicator to function as a shared analytical reference for identifying vulnerabilities in students’ existential health within pedagogical environments.

To ensure dependability, the progression from meaning units to candidate indicators was rigorously tracked through a systematic categorisation process. Researcher reflexivity was maintained through continuous dialogue between the two authors, whose diverse backgrounds in special education, public health, and pedagogy provided a form of investigator triangulation, reducing individual bias and enhancing the credibility of the manifest interpretations. Furthermore, by clarifying *new pedagogical definitions* for each health foundation (RQ2) from a pedagogical lens and from a sustainability perspective—connecting indicators at the individual level with the school level—important health-promoting aspects in a school context were identified. For example, school health-promoting aspects within the physical foundation highlight the importance of schools adopting a deliberate focus on daily physical activity to support students’ overall well-being. Additionally, concluding pedagogical definitions were developed regarding the manifestations and significance of children’s mental, physical, social, and existential health in a school context. These descriptions adopt an inclusive perspective. Accordingly, physical health is not defined merely in terms of performance, training, or the absence of injury or illness; rather, the concluding definition encompasses all children and young people in school, regardless of their physical disposition.

In the results section, which is descriptive in nature, similarities and differences in how children’s and students’ mental, physical, social, and existential health manifest in everyday school practices**—**from preschool through primary and upper-secondary school**—**are presented. The findings intentionally reflect health manifestations across different school forms and ages, which may lead to certain overlaps in order to capture the nuances of each specific educational context. The concluding section introduces a holistic perspective of *Pedagogical Health in School (PHIS).*

Finally, based on the current problematic situation, including alarming health issues among the younger generation, the article discusses the findings. From a pedagogical lens, PHIS may provide new insights into how this perspective can strengthen schools’ interprofessional capacity to promote children’s and students’ well-being, health, and growth, thereby contributing to sustainable educational practices.

### 4.3. Methodological limitations

While this study offers a novel perspective on school health promotion, several methodological limitations must be acknowledged. First, a recognised limitation is the exclusion of children’s and students’ own perspectives, Special Needs Schools and leisure centres. Due to available financial resources and time constraints, the data solely reflects the professional experiences and observations of school staff. Capturing the lived, experiential perspectives of students remains a crucial direction for future research to further validate and enrich the PHIS perspective. Second, the study was conducted within a single Swedish urban municipality. While the city’s high diversity and complex socioeconomic conditions provide an illustrative and important case for exploring children’s well-being, the findings should be viewed as context-sensitive examples rather than a universal checklist. Consequently, broader claims regarding international applicability should be made with caution, as the framework may require adaptation to different national and cultural educational systems. Finally, the analysis was deliberately bounded at a manifest level of interpretation to preserve the professional validity and immediacy of the candidate indicators for practitioners. While this choice supports the study's aim of developing shared, practice-recognisable reference points, it does not explore deeper latent meanings or subjective student experiences, which is a task for future empirical exploration.

## 5. Results

In the interviews, school staff describe their experiences of working with health promotion. A consistent picture emerges in which the participants testify to increasing health problems among children and young people in recent years. At the same time, the staff raise the issue that it is difficult to articulate what health-promoting work in school can entail. A preschool teacher puts it this way: “Well, we don’t talk about health promotion in those terms. We don’t. But I think that what we are working with is indeed aimed at promoting health”.

The analysis identifies 52 *candidate indicators* that encompass children’s and students’ health and ill health across the four foundations. The presentation of the empirical data is followed by a summary of candidate indicators at the individual level identified in the analysis. In the conclusion, key health-promoting aspects at the school level are summarised. Finally, these findings inform the new proposed pedagogical definition of health in a school context presented.

### 5.1. Mental health

The participants confirm the increase in mental health issues among children and young people. In line with this trend, the staff draw attention to current national guidelines that also directly concern the schools’ health-promoting work, which involves all staff.

We see poor mental well-being in many students, and I’m not sure whether it’s due to society. It’s a bit difficult to answer. I have been a principal for 15 years, and during the first 10 years, I didn’t notice any of this poor mental health. But then, at some point, it just increased. Students with depression diagnoses started coming from primary school. I had never seen that before, never ever, and since then, I feel it has only increased. *(Principal compulsory school)*

In Sweden, suicide prevention work has been highlighted. Then we have worked with that and emphasised that it is about preventing suicide… But it is also about working with mental health. We do that every day. It is important that student health work includes all the staff at the school. Because it is all the way into the classroom, all the time. That you have good treatment, that you can work preventively against conflicts and against insecurity. And that you also can act at an early stage. *(Teacher preschool)*

Based on their experiences, the staff describe concrete factors that contribute to students’ poor mental health. One common factor is growing up in economically disadvantaged areas. Moreover, several principals emphasise that the school can serve as a safe place for children living in vulnerable life situations.

To get food when you come to school. Not everyone has money. And the social aspect, with problems at home—getting away from home. School is a safe place for many children. *(Principal compulsory school)*

We also have people from economically weaker areas, but primarily from here, the areas are strong, and then there’s a lot of performance pressure and anxiety, as well as about prospects and capacity. (*Counsellor compulsory school*)

In the interprofessional discussions, the staff collectively highlight specific aspects such as anxiety and pressure, as well as the negative impact of social media, which can contribute to poor health, either individually or collectively.

At upper secondary school, the problems with anxiety, performance anxiety, and eating disorders are very evident—eating disorders of all different kinds. Self-harming behaviours. A lot happens on social media… bullying on social platforms. *(Teacher upper secondary school)*

One catches much more easily this outward behaviour. But when we look at mental health of students who do not eat, reaching this group who turn inward… It is about children who mask difficulties… Linked to anxiety and depression that is not noticed so clearly. *(Psychologist compulsory school)*

I think that upper secondary school students have become addicted to their phones. *(Teacher compulsory school)*

Many schools continuously engage in discussions and conduct surveys to capture students’ well-being and identify factors that may contribute to stress and mental illness.

A survey was conducted for all students in Year 2, focusing on mental health and well-being. The survey included different parameters and showed that a large majority of the students experienced future-related stress—that is, stress related to thoughts about the future. Linked to—*What will I do later?* Also perceived demands on themselves to perform, both in school subjects and in sports or other activities. *(Psychologist upper secondary school)*

What is the difference between feeling a little down and being depressed? *Where can I get support if I were depressed? How do I support a friend who is feeling bad?* Sometimes, the answer is not by keeping a secret. This is the kind of dilemma that many young people face, and it can lead to a very meaningful discussion. *(Psychologist upper secondary school)*

Such discussions are essential, as the relationship between students’ emotions and their manifestation across different school contexts may be challenging for young people to comprehend and regulate.

When students come into the classroom carrying a lot with them—conflicts, mental health issues—their learning drops drastically. If you feel bad in the classroom, you will feel bad overall*. (SEN teacher compulsory school)*

Consequently, if a young person experiences discomfort in the classroom, their overall well-being is compromised. As an essential aspect of mental health, the participants emphasise the importance of secure relationships between children, students, and adults in the school. School attendance constitutes another fundamental aspect.

How we can bring them into the classroom and get them to stay, and how health-promoting it would be for their development and for well-being in general. Our absence groups work with routines and structure for our students based on the insight that it is important for their mental health. *(SEN teacher compulsory school)*

Relationship building is incredibly important for promoting mental health. I feel that I can do much more for the individual child. I don’t have other obligations, so I can focus on a child who is struggling. I can work in a health-promoting way. *(SEN teacher preschool)*

In conclusion, the results reveal a complex picture of multiple candidate indicators within a school context that influence students’ mental health issues and overall well-being.

#### 5.1.1. Candidate indicators on mental health in school

Demands, feelings, stress, future-related stress, insecurity, anxiety, performance anxiety, depression, safety, money, food, eating disorders, self-harming behaviours, addiction, loneliness, meaningful relationships.

### 5.2. Physical health

When staff share their experiences on physical health, a strong emphasis is placed on ensuring children’s safety. The pedagogues testify that the deliberate focus on preventing harm to children has intensified in recent decades.

We cannot let the children come to harm in any way. If we have a planned lesson or something and there aren’t enough staff, then maybe we must skip that lesson. We might need to do something else with the children or find another solution. The children’s safety must always come first. *(Teacher preschool)*

According to current standards, safety is prioritised. In alignment with the curriculum, teachers describe their broad mission regarding the school’s health-promoting work, which encompasses various aspects, including movement, motor skills, diet, sleep and play.

Movement and diet. We do have our mission, of course—to follow the curriculum. Where it is also written that movement, motor skills, and diet support good development. This is then a part of the health-promoting work. (*Teacher preschool)*

I’m thinking about the whole picture. Why health is important, for example, sleep and diet, but also sports and the physical aspect. Helping the children realise for themselves that the more you move, the more energy you have to sit and work. For instance, we have organised recess activities during every lunch break. One person works with this, and it’s very much appreciated. They set up activities and movement-based things the children can do during break. It’s important, during their break, to move and play. *(Teacher compulsory school)*

The pedagogues emphasise the importance of children participating in pedagogical meals and in daily physical activities, where they play and develop different motor skills, ultimately outdoor time is important. However, staff previously did not frame these aspects as part of sustainable health-promoting work.

I want to highlight that when we now work with health-promotion, we also review the learning environments. For example, the preschool outdoor environments, to include physical activity. We consciously bring in those perspectives in this work… There should be outdoor time every day to meet the need for movement. We go outside every day, because it wouldn’t be health-promoting to remain inside all day. But we haven’t really approached health from a sustainable perspective. *(Teacher preschool)*

Teachers in compulsory and upper secondary school bear witness to the fact that teachers today do not have a clear focus on physical activity that was evident a decade ago and note that today’s young people are becoming progressively less physically active in school.

I feel that right now there is more emphasis on mental health for us teachers. If we go back, say 15 years, it was more about physical health. The health-promotion back then was about going outside. I remember going out with classes in both lower and upper grades. We were supposed to have 30 minutes’ walk a day, but then it was stopped. Now it’s more about mental health that people talk about. It’s as if physical health has become something you must take responsibility for yourself. *(Teacher compulsory school)*

We work a lot with movement breaks during lessons, to help the children understand how important that is… They are planned into my schedule. I have those moments when I know the lesson blocks will be long, so I’ve already included them. They can also happen spontaneously if you notice that the students are getting a bit tired. It’s also important that they learn the difference: talking about when it’s important to calm down and relax, and when it’s important to move. *(Teacher compulsory school)*

We don’t have any elements of movement. I think it’s partly about savings, because it does cost. You need staff, and that costs. It’s an organisational issue, since it takes staff time. We have a tight budget. *(Principal compulsory school)*

The results confirm the long-term trend of declining physical activity in schools. Another concern is that physical education in school is a subject that remains strongly oriented toward performance and grading, which often causes discomfort among many students. This corresponds with the observed decline in physical activities during breaks, as students instead spend time on social media.

At upper secondary school, the problem is less physical activity. Young children go outside during breaks and play in the schoolyard. Our teenagers sit and show each other TikTok and Instagram. They communicate through social channels. *(Teacher upper secondary school)*

The results indicate a trend of declining physical activity in schools among today’s younger generation. Moreover, the reduced emphasis on physical activity in schools necessitates increased staff awareness to counteract this sedentary trend and actively promote physical health.

#### 5.2.1. Candidate indicators on physical health in school

Movement needs, motor skills, physical activity, sports, safety, relaxation, sleep, diet, play, breaks, outdoor time.

### 5.3. Social health

In the problematisation of what health promotion can be, all participants emphasise the significance of social relationships between staff and children and students, as well as among students and children themselves.

Health-promoting work is broad. One might not be able to say, ‘use these methods’ or specifically ‘these interventions’. However, we have the children, and the youngest start when they are one year old. One could say that everything we do is health-promoting in some way. Because it is about attachment, it is about relationships, and a close interaction between educator-child and child-child. *(Teacher preschool)*

Play has a health-promoting purpose among social relationships. You might enter a role-play and imagine yourself in another role; when you talk, it is social connection. You feel good in that situation. *(Teacher preschool)*

We are not talking about health-promoting in those terms. But I think that what we work with is with the purpose of promoting health. Everyone in the team does a mapping to catch each child… This child is often alone, but what is the child interested in? We need to find a context where the child is together with someone. Then we evaluate it. We experience that the child feels better. I see that as psychological and social health-promotion. I think that such small health-promoting efforts are done all the time that one might not think about. *(SEN teacher preschool)*

In preschool, attachment, social connection, play, togetherness, and loneliness, emerge as essential aspects of creating safe and trusting relationships between teachers and children, as well as among peers. Furthermore, with an increased focus on teaching, the teacher-student relationship emerges as fundamental for addressing students and their diverse needs.

The relationship is the most important, it is number one. If the relationship does not exist, you cannot build on anything else. If the relationship exists, it does not matter how long it takes, but slowly but surely you will reach the student. If you create relations things won’t become as chaotic in the classroom. You build those positive relationships, you can listen properly and give good feedback, and you can bring the group together. *(Teacher compulsory school)*

Health in the classroom… that one is part of a context, that one reasons together, and help each other. The teachers call it *a way of teaching.* But if… one could notice what health-promoting measures a certain type of teaching entails. Similarly, relationship-building, what do we do and what is important? It is really an exciting thought to raise awareness among the teachers—that they find a form for what they do, every day in the meeting with their students. When they build relationships in different ways. When they give students support so that they succeed. Teachers say—I always do that. But when you ask a teacher, ‘What is it that makes your students thrive?’ They do not know. They have no insight into it. I think we need to talk more about it. What is it that makes one succeed in the classroom, to reach the students. Relationships in all types of schools are super important. *(Principal compulsory school)*

Based on successful experiences, principals emphasise the importance of interprofessional collaboration and highlight how crucial the classroom teacher is. Additionally, teachers describe the significance of collaborative work, mapping that all students have a relationship with an adult.

We work a lot relationally because we must start creating relationships. Something that has proven successful is that we have mapped all the relationships. We took class lists and worked with colour codes just to see if there is any student that no one has a relationship with… There were a few who, ‘No, I never talk to that student’. How can we approach and create trust? Then we have seen very good response. Both our students and guardians say that ‘we see ourselves seen’. This is a success factor, because we all know, if we have a relationship with a person, the risk is low that a student will be excluded. *(Principal compulsory school)*

We do have a student health service with a career counsellor, psychologist, school nurse, SEN teacher, and counsellor who are responsible for the students’ health. But the biggest work we do is still in relation with the teachers. That collaboration with teachers who have a relationship with individual students is almost the most important. *(Principal compulsory school)*

There is also an awareness among staff that schools receive new students and transfer students every year, which requires continuous and active relationship-building. Teachers also bear witness; on the one hand, today’s youth seek social contact, yet on the other hand, they do not socialise with peers to the same extent during breaks.

Most adults are good at building relationships, because that will be the most important to make the students feel that they always have someone at school they can talk to. I think we are good at building, building, building relationships*. (Counsellor upper secondary school)*

I think overall that the students are very good at making contact. They are seeking adults. They need a lot of confirmation, and they seek confirmation in different ways. Not all, but many, students are very contact seeking, want to talk about different things. *(SEN teacher upper secondary school).*

Being a teenager and sitting and hanging out in the corridor and chatting with friends doesn’t exist. *(Career and study counsellor)*

Another essential relational aspect concerns contact with guardians. The results show how trusting relationships lower the threshold, enabling guardians and students to seek help early, for example in cases of school absenteeism.

Work with the relationships much earlier and create good relationships with the guardians to be able to raise the difficult questions when the absence spikes. We want to have the child in school even if it’s just for a short while. Just to get the habit, so I must work with the parents with the relationships. You can offer a lot of parent education. But at the end of the day, I think it is the relationship that is decisive for us to get the student back. *(Principal compulsory school)*

To conclude, the importance of safeguarding relationships applies to both children and adults and is evident within the school climate. Secure and trusting relationships constitute the foundation of a healthy working environment for both students and staff.

#### 5.3.1. Candidate indicators on social relationships in school

Meaningful teacher-student relationship, peer relations, pedagogical encounters, relationality, attachment, contact seeking, social interaction, connection, someone to talk to, trust, togetherness, friends.

### 5.4. Existential health

Understanding students’ health also requires attention to existential concerns regarding individual beings and their sense of belonging within the school community, as well as recognising children’s right to health.

You have a group of children, but you also have individuals. They are all different individuals. One child has one need another child has a different need. Everyone has the right to their health. *(Teacher preschool)*

Health can mean so many things. Health is about coming to school. It’s also preventive for adult life… It’s about being allowed to be someone. To achieve and succeed, but also simply to be allowed to exist. I think it’s equally about being part of a community. To be allowed to be a person within a context, to have one’s voice heard and to be seen—that, too, is part of health. It’s also about being given the opportunity to reason and reflect on different things related to health. It’s about becoming part of something—that the students become visible to the teachers, and that the teachers dare to engage with the students’ thoughts. I can see that our upper secondary students have so many thoughts… Media and TV bring up so many questions. Fear, for instance—what if there’s a war here? Students need to express and process those thoughts. They need to meet adults who can listen, and then they can calm down again*. (Principal compulsory school)*

How does one seek help? What help and support are available? Who is the school counsellor? Who is the school nurse? Who conducts the health talks? Our psychologists also offer support sessions in upper secondary school. Then we can also work with a sense of coherence. *(School counsellor upper secondary school)*

The results highlight existential aspects such as existence, sense of coherence, being seen and heard, and having the opportunity to speak with unique voices. Furthermore, today’s school maintains a clear focus on performance and offers few opportunities for rest and social interaction, which fosters fear of failure and anxiety.

How do we view children? How do we talk about children? We make a difference when more children dare to ask for things and speak up. Self-esteem. Adults who see me and hear me? *(SEN teacher preschool)*

Much of what young people do today is about performance. The feeling of being judged in many different contexts, at school and during their free time… It’s something you can’t really get away from nowadays… The matter of daring to speak is closely linked to the fear of failing. I think it’s important to get the chance to practice and maybe feel that it’s OK to fail. *(Teacher compulsory school)*

There are so many students who feel they must be the best at everything. We have a lot of high achievers, but that doesn’t mean they’re all happy with it. It’s a real challenge to help them deal with that stress. They get this kind of tunnel vision, like they must excel in everything… and it ends up turning into an identity crisis. *(Career counsellor upper secondary school)*

The high demands that individuals place on themselves, both in school and in today’s society, raise existential questions of identity concerning who you are and why you attend school.

Anxiety issues and worry, a feeling of exclusion. A lack of a sense of belonging. It’s largely about that. They don’t really know why they are here in school. (*Counsellor compulsory school*)

In today’s society, you can’t really be yourself. You must keep up a mask… If you’re not a certain way, you’re not accepted. That scares me. I wonder… what will it look like in the future. (*SEN teacher compulsory school)*

Every little thing you do feels huge. Every decision seems to matter so much. Should I grab a pizza with my friends, or should I go to the gym? No—my whole future depends on this. *(Counsellor upper secondary school)*

Beyond measurable aspects of health, the existential foundation also highlights how young people make sense of their lives, addressing questions about pressure, violence, safety, life and death. This finding underscores the importance of how staff can support students in achieving balance in life.

Our social pedagogues work socially and relationally investigating violations, but above all promoting attendance… Somewhere we must constantly land in our core mission to educate. This is school and we work with education. To succeed, we must have students who feel that they are well and have the opportunity to learn things. It is important that students feel safe. *(Principal compulsory school)*

I want all the time to become better at taking in the students’ needs. I think that we need to become better at listening to them. How would they want it to be arranged so that it becomes good for them... We also have some exercises about resilience, presence and alternative thoughts. *(Psychologist upper secondary school)*

Young people… there’s been an inflation in words. If you have a headache, then you have a migraine. If you’re sad or worried, then you have anxiety. We need to teach children and young people that life is quite tough… It’s awful to fail a test, and when someone breaks up with you and you end up alone… We need to strengthen and support them in that. Because later, the tough things will come. We can talk about everyday life—the things about life’s ups and downs. We can have values-based work with certain questions that are brought up. A whole-school approach that is worked with every week where there is a forum. You sit down and talk about what has been good during the week. It is an open conversation about what feels good. It is a health aspect—to be present and to feel that you are safe and comfortable. *(Teacher compulsory school)*

To summarise, the staff highlight the significance of working with students by listening to their existential reflections, discussing life’s fluctuations, and supporting them in developing alternative tools during challenging times. Consequently, such health-promoting work requires value-based forums with an open safe climate.

#### 5.4.1. Candidate indicators on existential health in school

Unique children, being in the world, be yourself, daring to speak with your unique voice, identity, self-esteem, existential thoughts (why do I exist, why am I in school), belonging, sense of coherence, pressure, being judged, resilience, alternative thoughts when life is hard.

## 6. Conclusion - Pedagogical Health in School (PHIS)

### 6.1. Health candidate indicators

Through a *pedagogical lens* on the educational environment (Ljungblad, 2021), the analysis identified *52 candidate indicators* that can be used by all school staff to detect factors that promote or undermine children’s and students’ health and well-being in schools. To provide a comprehensive overview of these empirical findings, Table II presents the candidate indicators categorised within the *four health foundations.* These candidate indicators represent *practice-recognisable expressions* of health and ill health, serving as a *shared conceptual base* for interprofessional collaboration in schools. At the individual level, these candidate indicators form unique and shifting profiles for each child that may change over the course of their schooling. Consequently, these unique profiles can guide student health teams and teachers in their work with each child, providing a pedagogically sensitive way to follow and support each student’s evolving health pathway.

**Table II.** Candidate indicators that signal children’s and students’ health and ill health across the four health foundations.

Health foundations	Health candidate indicators
Mental Health	Demands, feelings, stress, future-related stress, insecurity, anxiety, performance anxiety, depression, safety, money, food, eating disorders, self-harming behaviours, addiction, loneliness, meaningful relationships.
Physical Health	Movement needs, motor skills, physical activity, sports, safety, relaxation, sleep, diet, play, breaks, outdoor time.
Social Health	Meaningful teacher-student relationship, peer relations, pedagogical encounters, relationality, attachment, contact seeking, social interaction, connection, someone to talk to, trust, togetherness, friends.
Existential Health	Unique children, being in the world, be yourself, daring to speak with your unique voice, identity, self-esteem, existential thoughts (why do I exist, why am I in school), belonging, sense of coherence, pressure, being judged, resilience, alternative thoughts when life is hard.

### 6.2. School health-promoting work

To bridge the gap between individual-level health expressions and school-level organisational structures, the identified candidate indicators function as *second-order constructs* that translate broad health concepts into practice-recognisable units. These indicators inform how schools can systematically direct their focus towards health-promoting work, providing a *shared professional language* for staff to identify, discuss, and act upon well-being within daily pedagogical life. By aligning professional observations with organisational actions, this approach enables schools to implement sustainable health promotion that is directly grounded in the children’s lived experiences.

First, schools play a key role in supporting *mental health* by providing safe, structured, and inclusive school environments with preventive work against conflict and bullying. Through the lens of pedagogy, health-promoting work includes an understanding of children and students’ feelings of stress, demands and performance which may present as introverted or extroverted behavioural patterns. School staff therefore need to understand and handle students’ perceived emotions in various school situations.

Second, in the *physical health*-promoting pedagogical approach to physical well-being, schools face the challenge of balancing two important aspects: on the one hand, fostering children’s and young people’s curiosity and exploration of new physical activities, and on the other hand, ensuring safety and minimising the risk of injury. Schools’ health-promoting work regarding physical health also includes ensuring that students gain knowledge about the balance between diet, activity, rest, sleep, and recovery. Moreover, schools need to adopt a deliberate focus on daily physical movement to support students’ overall well-being. From a pedagogical perspective, it is also essential for schools to create safe and inclusive learning environments for sports activities, while finding an appropriate balance between play, participation, and assessment.

Third, a *social health*-promoting approach to social relationships includes teacher-student relationships, peer relations, and relationships with guardians—a responsibility shared by school staff. Schools require collaborative, interprofessional efforts in which staff, from a health-promotion perspective, systematically map relationships, design interventions, and evaluate dimensions of participation, safety and trust. Creating trustful relationships requires presence, active listening, and helpful feedback, which together cultivate healthy and sustainable social relationships.

Fourth, from an *existential health*-promoting perspective, schools need to address existential aspects in education that support both emotional well-being and academic engagement. Engaging with young people’s existential questions involves fundamental dimensions of being heard and seen throughout life’s ups and downs. From a pedagogical perspective, schools can foster an open climate where students know how to seek support from various professionals and are encouraged to develop alternative ways of thinking during challenging times.

### 6.3. Concluding pedagogical definitions

To further bridge the gap between high-level policy and everyday school life, the following pedagogical definitions offer a concrete base for both practitioners and policymakers in their health-promoting work. By synthesising broad health concepts with the empirically derived candidate indicators, these definitions provide the essential structure for schools to implement systematic health promotion that is sustainably integrated into both the curriculum and organisational policy. This approach ensures that local educational efforts remain grounded in the children’s lived experiences while being directly aligned with international standards such as the WHO and UNESCO’s Health Promoting Schools framework and the United Nations Sustainable Development Goals. Based on the identified health candidate indicators, the following pedagogical definitions are proposed for each foundation.

*Mental health* in a school context refers to the well-being of children and students, enabling them to understand and manage emotions and participate in meaningful relationships in the classroom. From a pedagogical perspective, the school staff need to provide continuous support so that children and students can develop their strategies and tools to cope with stress and performance in a balanced way.

Young people’s *physical health* within the school environment encompasses movement ability in relation to individual capacities and safety concerns. This is approached with a pedagogical focus on balancing play and participation rather than performance, and on supporting children’s and students’ ability to move according to their own capacities, thereby creating positive experiences of physical health.

In the school environment, a trustful interpersonal relationship between the teacher and the child/student is fundamental to *social health*. Through daily pedagogical encounters and face-to-face interactions, teachers have the opportunity to see each child and explore—*Who* are you? From a pedagogical perspective, a trustful teacher-student relationship is essential for the social well-being and growth of individual children.

Schools encounter all children and students and play a crucial role in recognising and supporting their *existential health.* Creating a safe and open classroom environment allows children to express their thoughts and emotions freely. From a pedagogical perspective, school staff need to address young people’s existential questions about life, meaning, and values, helping them navigate identity, build resilience, and develop tools to face life’s challenges.

### 6.4. Pedagogical health in school - a holistic, pedagogically grounded perspective

The exploration of the umbrella concept *pedagogical health* lays the foundation for a holistic pedagogically pragmatic, grounded perspective on health based on the child’s right to health (United Nations General Assembley, 1989). Building on pedagogical theory and ecological perspectives on human development (Bronfenbrenner, 1979; Seligman, 2011), pedagogical health is positioned as a holistic concept that integrates the mental, physical, social, and existential foundations of children’s and students’ health in school, which also support theory development. These foundations are understood as interdependent and embedded within the pedagogical processes and relationships that shape everyday educational environments.

Furthermore, four strategic focus areas for school health-promoting work (see 6.2) and the proposed pedagogical definitions within each health foundation (see 6.3)—together connecting the school level with the individual level—were established. Together, the findings show that *pedagogical health* provides a vital sustainability perspective for understanding and addressing the complex conditions shaping children’s and students’ lives in today’s schools and supports schools in aligning their health-promoting practices (WHO & UNESCO, 2021). Figure 1 presents a conceptual model of *Pedagogical Health in School (PHIS),* encompassing the four foundations—mental, physical, social and existential health—including their candidate indicators, understood as an integrated whole.

**Figure 1.**
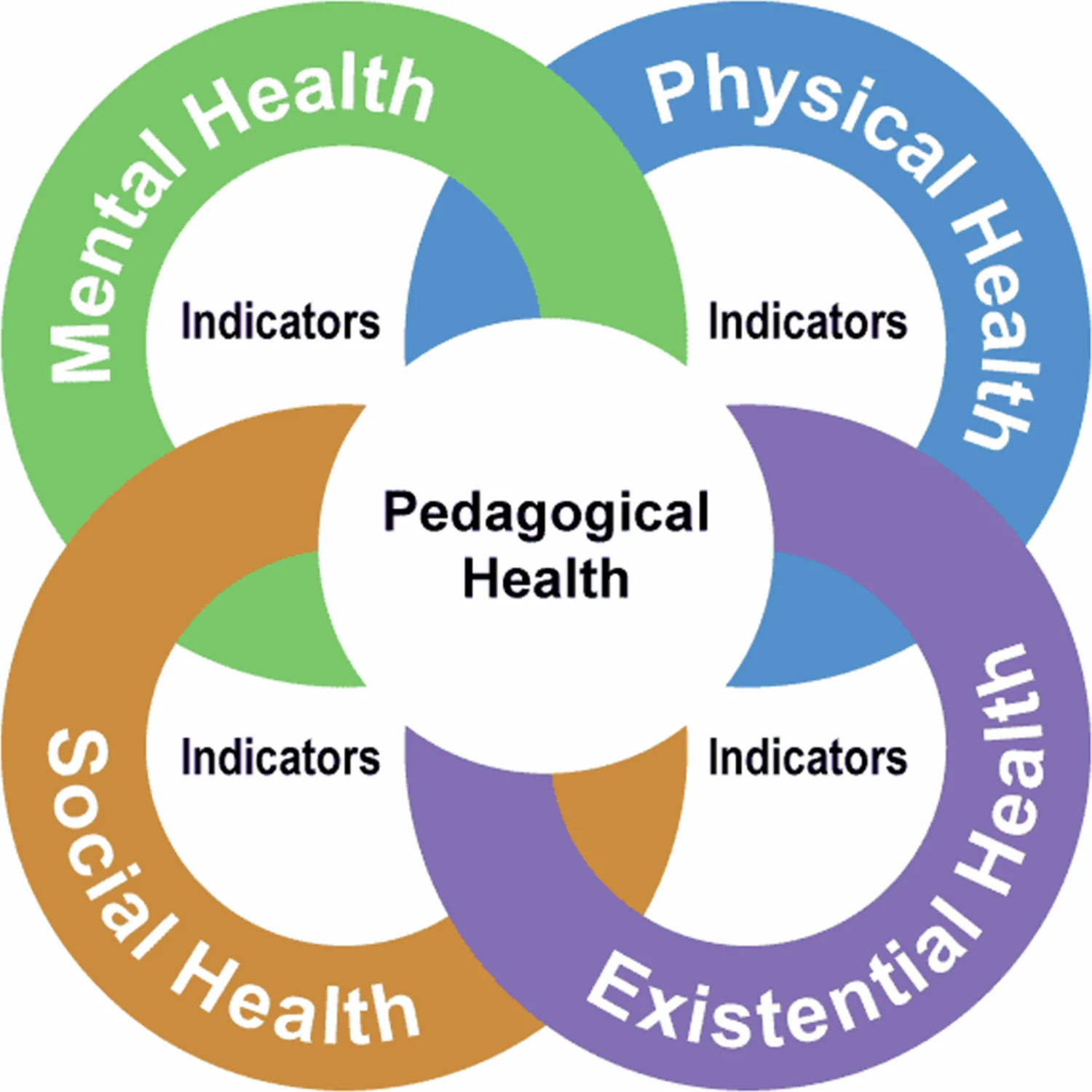
A conceptual model of Pedagogical Health in School (PHIS) encompassing the four foundations of children’s and students’ mental, physical, social, and existential health.

The conceptual model highlights the pedagogical nature of health in school by linking health-related conditions to intentional educational actions and to students’ opportunities for participation, meaning-making, and growth. Since PHIS provides a shared conceptual base, the following definition of *pedagogical health* is proposed, which includes both health promotion and health education.

*Pedagogical health* encompasses the mental, physical, social, and existential health of children and students within a school context. The concept integrates both school health-promotion work through intentional actions and students’ health education grounded in health didactics. As such, pedagogical health reflects a holistic and sustainable approach in which the educational environment actively supports well-being, participation, personal development, and academic achievement.

From a pedagogical lens, the theoretical framing of PHIS offers a potential for developing a shared language (Beckman et al., 2023; Choi, 2024) and common understandings across professional groups in schools, providing a basis for future work on sustainable, pedagogically grounded health-promotion practices. By reflecting the complexity of contemporary educational environments and the lived experiences of today’s younger generation, PHIS establishes a conceptual structure that can guide further theoretical refinement and empirical exploration of pedagogical health in diverse global educational contexts.

## 7. Discussion

The alarming rise in health problems among today’s younger generation underscores the urgent need for schools to adopt more coherent and sustainable strategies for promoting well-being (Cosma et al., 2023; United Nations Department of Economic & Social Affairs, 2025; World Health Organisation, 2025a). The widening gap between young people’s needs and the capacity of educational institutions to respond has profound implications for individual life trajectories, family wellbeing, and broader social equity. There is a critical need for decisive action to mitigate the growing impact of health challenges on future generations and to ensure that vulnerable children and adolescents worldwide can reach their full potential (Kieling et al., 2011). A whole-school approach is central to strengthening schools’ capacity to support children’s health and development in nuanced and context-sensitive ways (WHO & UNESCO, 2021).

Exploring *Pedagogical Health in School* highlights its potential as a practically grounded perspective that can enhance schools’ ability to work systematically with health promotion across professional boundaries and to develop sustainable, health-promoting practices (Edwards et al., 2024). While schools constitute a unique pedagogical arena that reaches all children, the complexity lies in how they can create opportunities for each child to emerge, express their own voice, and flourish (Ljungblad, 2021). Building on this complexity, it becomes essential to consider how schools can organise their health-promotion work in ways that honour each child’s unique developmental and wellbeing path (Langford et al., 2015). Rather than replacing existing programmes or models, PHIS has the potential to be integrated into prevailing health-promotion initiatives by offering a *pedagogically sensitive lens* that attends to each child’s unique health profile and supports continuity across the entire school health pathway. This allows schools to follow children’s evolving health conditions over time while maintaining coherence between individual support and school-level practices.

A pedagogical health perspective provides a unifying ground where different health and education professionals can collaborate, jointly follow each student’s evolving health pathway, and integrate well-being into everyday school practice. Such a health-promotive perspective enables schools to move beyond isolated or short-term interventions and instead cultivate structures that foster participation, inclusion, and long-term wellbeing. Interprofessional collaboration is therefore crucial: when teachers, school nurses, counsellors, social workers, and other professionals work toward shared goals, they develop a richer understanding of students’ diverse conditions and can design more coordinated and equitable strategies. Consequently, early-stage interventions play a particularly important role, yet further research is needed to understand which intervention characteristics ensure long-term sustainability (Simovska & McNamara, 2015; Spencer et al., 2025).

To conclude, we underscore the importance of proactive, sustainable, and pedagogically grounded approaches to child and adolescent health. This includes supporting students throughout their entire school trajectory to ensure continuity, equity, and long-term efficiency in health promotion—an area where pedagogical health may contribute to integrating wellbeing and growth in more meaningful and sustainable ways. To conclude, *Pedagogical Health in School* represents an approach aligned with the United Nations Sustainable Development Goals—particularly *SDG 3 (Good Health and Well-being)* and *SDG 4 (Quality Education)*.

Ultimately, this study contributes to a global conversation by introducing the novel perspective of *Pedagogical Health in School (PHIS).* Serving as a pedagogical lens that bridges the individual lived experiences of children’s health by introducing 52 candidate indicators with school-level structures, PHIS provides a shared conceptual base for interprofessional collaboration, thereby strengthening the capacity of schools to foster proactive and sustainable well-being for every student. Finally, we welcome future international studies that incorporate the lived experiences and voices of children and students, and parents, as well as professionals from diverse educational and cultural settings. Such perspectives are essential to validating, expanding and deepening our global understanding of how pedagogical health can be effectively promoted and sustained within diverse school settings.

## Data Availability

Due to the qualitative nature of this study and to protect the privacy and full confidentiality of the participants, the data supporting the findings of this research are not publicly available and cannot be shared.
